# Localized chemotherapy approaches and advanced drug delivery strategies: a step forward in the treatment of peritoneal carcinomatosis from ovarian cancer

**DOI:** 10.3389/fonc.2023.1125868

**Published:** 2023-05-23

**Authors:** Silvia Breusa, Serena Zilio, Giuseppina Catania, Naoual Bakrin, David Kryza, Giovanna Lollo

**Affiliations:** ^1^ Univ Lyon, Université Claude Bernard Lyon 1, Centre National de la Recherche Scientifique (CNRS), LAGEPP Unité Mixte de Recherche (UMR) 5007, Villeurbanne, France; ^2^ Apoptosis, Cancer and Development Laboratory- Equipe labellisée ‘La Ligue’, LabEx DEVweCAN, Institut PLAsCAN, Centre de Recherche en Cancérologie de Lyon, Institut national de santé et de la recherche médicale (INSERM) U1052-Centre National de la Recherche Scientifique - Unité Mixte de Recherche (CNRS UMR)5286, Université de Lyon, Centre Léon Bérard, Lyon, France; ^3^ Sociétés d'Accélération du Transfert de Technologies (SATT) Ouest Valorisation, Rennes, France; ^4^ Department of Surgical Oncology, Hospices Civils de Lyon, Centre Hospitalier Lyon-Sud, Lyon, France; ^5^ Centre pour l'Innovation en Cancérologie de Lyon (CICLY), Claude Bernard University Lyon 1, Lyon, France; ^6^ Imthernat Plateform, Hospices Civils de Lyon, Lyon, France

**Keywords:** peritoneal carcinomatosis (PC), ovarian cancer, PIPAC technique, nanomedicine, cancer treatment

## Abstract

Peritoneal carcinomatosis (PC) is a common outcome of epithelial ovarian carcinoma and is the leading cause of death for these patients. Tumor location, extent, peculiarities of the microenvironment, and the development of drug resistance are the main challenges that need to be addressed to improve therapeutic outcome. The development of new procedures such as HIPEC (Hyperthermic Intraperitoneal Chemotherapy) and PIPAC (Pressurized Intraperitoneal Aerosol Chemotherapy) have enabled locoregional delivery of chemotherapeutics, while the increasingly efficient design and development of advanced drug delivery micro and nanosystems are helping to promote tumor targeting and penetration and to reduce the side effects associated with systemic chemotherapy administration. The possibility of combining drug-loaded carriers with delivery via HIPEC and PIPAC represents a powerful tool to improve treatment efficacy, and this possibility has recently begun to be explored. This review will discuss the latest advances in the treatment of PC derived from ovarian cancer, with a focus on the potential of PIPAC and nanoparticles in terms of their application to develop new therapeutic strategies and future prospects.

## 1 Introduction

With 295,000 new cases and 184,000 deaths worldwide in 2018^1^ ovarian carcinoma, and its most common form epithelial ovarian carcinoma (EOC), is the leading cause of death among gynecologic malignancies. Despite a high response rate to initial treatment (1), most patients develop disease recurrence within 2 years. The abdominal cavity and peritoneum are the sites most involved in the metastatic process that characterizes the advanced stages (stages III and IV) of ovarian cancer (2, 3). The five-year survival rate of ovarian carcinoma is close to 45%, however, most of these cases refer to patients diagnosed in early stages (I and II) who have 5-year survival rates of 95 and 70%, respectively. Unfortunately, only a minority of patients are diagnosed early, and the 5-year survival rate of patients with stage III or IV primary ovarian cancer drops to 25 and 15%, respectively, with the exception of patients with mutations of the *BRCA* genes who show a better response to treatment^2^. These numbers highlight the poor efficacy of current therapies in treating this deadly disease and underscore the urgent need for additional and alternative therapeutic strategies.

In this review we will focus on the treatment of high-grade ovarian cancer and PC, exploring the potential of localized chemotherapy to improve drug delivery and tissue penetration. Insights will be provided on novel locoregional delivery systems already or possibly deliverable by pressurized nebulization such as PIPAC (Pressurized Intraperitoneal Aerosol Chemotherapy) and e-PIPAC (electro-Pressurized Intraperitoneal Aerosol Chemotherapy).

### 1.1 Development of peritoneal carcinomatosis in ovarian carcinoma

Due to late diagnosis and high heterogeneity in clinical behavior and biological properties, ovarian carcinoma is still one of the most lethal gynecological cancers (4). It exhibits extensive malignant progression, rapid development of drug resistance, and associated cross-resistance, which are major unresolved clinical problems.

Ovarian cancer exists in different histotypes depending on the type of cell that underwent the initial neoplastic mutation. More than 90% of ovarian tumors originate from the epithelial surface of the ovary, while the remaining 10% originate from the germ cells or stroma. EOC can be further identified as serous (68-71%), endometrioid (9-11%), mucinous (3%), clear cell (12-13%), and Malignant Brenner (1%) (5, 6). These subtypes differ in terms of risk factors, biological behavior, and response to treatment. Early diagnosis is hampered by the lack of appropriate tumor markers and the paucity of symptomatic manifestations until the advanced stage of the disease, therefore, most patients are diagnosed when the tumor has already spread to the abdominal area and the clinical outcome is already compromised (4).

EOC originates from the serous lining of the ovary, which is in close contact with the peritoneum, the serous lining of the abdomino-pelvic cavity. The process of deposition and colonization of cancer cells in the peritoneum is known as PC, a difficult-to-treat condition that often leads to recurrence and death. During the development of ovarian carcinoma, tumor cells may detach from the primary tumor site through a process called exfoliation, probably mediated by the downregulation of adhesion molecules, such as E-cadherin, on the surface of tumor cells (7) and facilitated by the high interstitial fluid pressure common to many solid tumors (8). These mechanisms have also been confirmed for colon (9) and gastric (10) cancer with peritoneal spread. Because of the anatomical location, gravity, peristaltic movement of the gastrointestinal tract, and negative pressure exerted by the movements of the muscles of the diaphragm, exfoliated cells commonly implant in the pelvic and subdiaphragmatic region, and their adhesion to the mesothelial layer of the peritoneum appears to be mediated by glycan-binding proteins expressed by mesothelial cells (11) and by adhesion molecules such as CD44, integrins, selectins, and a number of other leukocyte-associated adhesion molecules (12). Tumor cells then penetrate into the submesothelial basement and consequently into the subperitoneal tissue due to the contraction of mesothelial cells and the degradation of the peritoneal tissue (13) and to the degradation of the peritoneal blood barrier (14). Another possible route of peritoneal spread of cancer cells is by the transmesothelial route, in which cancer cells enter the subperitoneal lymphatic space through lymphatic stomata and milky spots (15), small structures composed of macrophages and lymphocytes that are in contact with the peritoneal membrane (16).

First-line chemotherapy for the treatment of ovarian cancer is administered systemically by intravenous (IV) infusion and is often the only option in most patients with multifocal progression in the peritoneum. Despite the high response rate to initial treatment, most patients develop disease recurrence within 2 years (1). The rationale for the use of intraperitoneal chemotherapy (IPC) stems from the observation that IV administered chemotherapy drugs have low concentrations in the peritoneum, regardless of peak serum values (17). In addition, the peritoneal cavity is identified as a virtually large area that can increase the spatial and temporal exposure of the tumor to the drugs, reducing the absorption of the drug into the systemic circulation and thus its toxicity. We will discuss these aspects later in this review.

### 1.2 Conventional therapeutic approaches for ovarian cancer and mechanism of resistance

Depending on the stage at the time of diagnosis, treatment of primary EOC may be limited to surgery or accompanied by chemotherapy and, in rare cases, radiation or immunotherapy. Cytoreduction surgery (CRS) is performed as first-line therapy in all stages of the tumor and includes hysterectomy, removal of the ovary, removal of the omentum, and any other site compatible with removal. PC occurs in EOC stages III and IV, when the tumor has spread outside the pelvis and lymph nodes but is still within the abdominal cavity (stage III) or has distal metastases (stage IV). In these patients, the outcome of CRS has prognostic value (18, 19), patients with optimal CRS (no residual lesion is > 1 cm) have a median survival of 39 months compared with 17 months for patients with suboptimal CRS (20). For stage III patients, combination of CRS with subsequent cycles of IV and/or intraperitoneal (IP) infused chemotherapy is the main option.

Several studies have shown that IV administration of DNA cross-linking drugs such as platinum derivatives induces improved response rates in patients with EOC (21, 22). Carboplatin is currently widely used in the clinic, as it has less severe side effects than cisplatin (23) and resulting in an overall improvement in patients’ quality of life (24–33). However, the development of platinum resistance is common in patients with advanced ovarian cancer. Platinum-sensitive patients who respond to the first-line chemotherapy regimen and relapse after 6 or more months have a response rate to subsequent platinum-based therapies ranging from 30 to 90% (34–37) but most of them will eventually develop platinum-resistant tumors. Patients who relapse within 6 months have a response rate to new chemotherapy of 15% and have a short progression-free survival interval (3-4 months) and a median survival of less than 1 year. Platinum resistance may be limited by the combination of taxanes (paclitaxel or docetaxel), a class of mitotic inhibitors that block cell proliferation by disrupting microtubule function. To date, carboplatin/taxane is the gold standard postoperative chemotherapy regimen worldwide, with clinical response rates > 60% and median time to recurrence usually > 1 year (23). Among taxanes, docetaxel and paclitaxel show similar efficacy and progression-free survival rates when combined with carboplatin (38). They also exhibit incomplete cross-resistance, and clinical trials have shown that docetaxel administration is effective in patients refractory to paclitaxel regimens (39). However, the 5-year survival rate of stage III and IV patients undergoing optimal CRS flanked by systemic chemotherapy is close to only 30% (39).

The emergence of platinum resistance is partly due to increased DNA repair due to the modification of key proteins associated with this mechanism (40, 41). An example of particular interest is the secondary mutation of the *BRCA1* and *BRCA2* genes that causes restoration of BRCA function and consequently reacquisition of DNA repair activity (42, 43). *BRCA1/2* function as tumor suppressor genes by playing an important role in DNA repair through homologous recombination (44–47). Approximately 15-20% of ovarian cancer patients have a germline mutation of *BRCA1/2* (48, 49). Due to the ineffectiveness of cancer cells to repair DNA damage these patients show a higher likelihood of responding to second-line platinum-based therapies than patients with wild-type *BRCA1/2* resulting in a more favorable clinical outcome and higher survival rate (50–53).

The observation of *BRCA* mutations as favorable prognostic factors led to the introduction, in 2014, of the use of poly (ADP-ribose polymerase) inhibitors (PARPi) (54). PARPi are a class of drugs systemically orally administered that, by competing with nicotinamide (NAD+) for the catalytic active site of PARP molecules, can exploit *BRCA* mutations and deficiencies in DNA damage response. PARPi induce propagation of DNA damage that cannot be repaired due to the inefficiency of *BRCA1/2* activity resulting in cell death. In 2017, after the significant improvement in progression-free survival achieved with PARPi in three randomized phase III trials: NOVA/ENGOT-OV16 (NCT01847274), SOLO-2/ENGOT-OV21 (NCT01874353) and ARIEL3 (NCT01968213) (55–57) the use of PARPi has been extended to maintenance therapy for platinum-sensitive relapsed primary ovarian, fallopian, and peritoneal cancers, regardless of *BRCA* status (56, 58, 59). To date, olaparib, rucaparib and niraparib are also approved as monotherapy for pretreated recurrent ovarian cancer (60).

### 1.3 The intra peritoneal path for the management of peritoneal carcinomatosis

Despite the progress made with the introduction of new drugs that can circumvent molecular-based drug resistance, the efficacy of these new therapeutic approaches in patients with peritoneal metastases is limited, suggesting that other mechanisms must be involved in the chemoresistance of these diseases (61). For example, high dosing is known to facilitate the onset of multiple drug resistance (62). In PC, high dosages of IV chemotherapy are necessary to achieve therapeutic efficacy since the presence of the peritoneal-plasma membrane prevents the passage of large molecules, and most drugs, from the bloodstream to the peritoneal cavity and vice versa (63).

However, the presence of the peritoneal-plasma membrane may be an advantage for the treatment of diseases limited to the peritoneal cavity, as an administration of chemotherapeutics directly into the peritoneum may reduce systemic toxicity (64–66). In PC, locoregional administration (IP) thus has the advantage of increasing drug concentration in the residual tumor, avoiding drug leakage and systemic adsorption, as initially demonstrated in 1978 by Dedrick and colleagues (67) and later validated by early clinical trials in which the IP route of administration showed a 10- to 20-fold higher dose of tumor chemotherapy than the IV route (17). The peritoneal-plasma barrier, consisting of the peritoneal mesothelium, subserosal tissue, and blood vessel walls, appears to be primarily responsible for maintaining high drug concentrations in the peritoneum (68–70) preventing the transfer of high molecular weight and hydrophilic drug molecules into the systemic circulation (71). Drugs administered to the peritoneum can also be adsorbed from the peritoneal cavity through the lymphatic vessels, and the hypothesis that this phenomenon may help treat retroperitoneal lymph node metastasis was demonstrated by a randomized subtrial that showed that the survival benefit of IP over IV chemotherapy in ovarian cancer was independent of the patient’s lymph node status (72).

Unfortunately, less drug penetration into the tissue stroma has been observed with IP administration via catheter compared with IV administration, and its application is beneficial only for patients in whom optimal CRS has been achieved. Furthermore, although median disease-free survival was increased with IP chemotherapy compared with IV chemotherapy (73) the IP route still retains high toxicity, as demonstrated in the GOG-172 clinical trial (NCT00003322) in which only 42% of patients receiving IP chemotherapy were able to complete their scheduled chemotherapy cycles. Most of the side effects recorded during this study were related to catheter-related problems, poor tolerance of IP treatment, complications of chemotherapy, or disease progression (74). An in-depth description and summary of these studies were comprehensively reviewed in (75).

Another problem related to catheter-IPC is that this procedure is usually performed weeks after CRS, when extensive adhesions have already developed in the peritoneal cavity as a postoperative consequence. Adhesions hinder the efficient distribution of IPC in the peritoneum, as they impair the ability of the drug solution to distribute properly in the abdomen.

Because of the problematic tolerability of IP chemotherapy administered via catheter, this approach has not been included in routine clinical practice; however, it has set the stage for the development of other techniques such as hyperthermic intraperitoneal chemotherapy (HIPEC) and pressurized intraperitoneal aerosol chemotherapy (PIPAC).

### 1.4 Locoregional treatments based on intra peritoneal administration: HIPEC and PIPAC

HIPEC consists of a single administration of heated chemotherapy solution onto the peritoneal surface of the abdomen and is usually performed immediately after CRS. The purpose of HIPEC is to eradicate microscopic foci of disease that cannot be surgically removed. Unlike catheter administered IPC, in the case of HIPEC, the perfusate is administered as an intraoperative treatment after CRS, prior to the development of adhesions and provides homogeneous exposure of the entire seroperitoneal surface to both drug and heat (70). In addition, the intraoperative combination of CRS and HIPEC allows immediate treatment of the residual tumor, facilitating its eradication and removing the need to install peritoneal access devices on patients, thus eliminating the resulting catheter-related complications (76). Other advantages that make HIPEC preferable to traditional IPC are related to the temperature of the drug solution (around 42°C). Hyperthermia has direct cytotoxic activity on tumor cells and shows a synergistic effect with many antiproliferative agents such as, cisplatin and oxaliplatin, paclitaxel, and mitomycin (77). In addition, typical hypoxic tumor cells are more sensitive than normal cells to hyperthermia (78) which also enhances the penetration ability of chemotherapeutics (79, 80) contributing to increasing the sensitivity of tumor cells to drug treatment (81–83). Hyperthermia has also been linked to an enhanced antitumor immune response through Heat Shock Proteins 90 (HSP90) (84) and to an increase in lymphocyte migration and activation of antigen-presenting cells (85, 86).

Until recently, global acceptance of HIPEC has been hampered by a lack of solid evidence of efficacy, as promising data were mainly derived from small case series, nonrandomized comparative studies, and systematic reviews (87–95). In addition, these studies are not homogeneous in terms of timing of administration, disease status (primary or recurrent) active molecules, and dosage used. HIPEC performance is expected to be optimal when administered for the treatment of chemosensitive tumors both at the beginning of treatment course or as consolidation therapy, thus it can be strongly influenced from these differences. In addition, because of the high heterogeneity of ovarian cancer patients, the lack of randomization has been a major limitation. To date, there are 14 ongoing international randomized phase 3 trials investigating the use of HIPEC in the treatment of women with ovarian cancer at different time points (**Table 1**).

**Table 1:** Clinical trials involving the use of HIPEC for the treatment of disseminated peritoneal ovarian carcinoma.

Title	Identifier	Status	Conditions	Patients	Interventions	Drug
Hyperthermic Intra-Peritoneal Chemotherapy (HIPEC) in Relapse Ovarian Cancer Treatment (CHIPOR)	NCT01376752	Active, not recruiting	Recurrent Epithelial Ovarian Cancer	415	Maximal cytoreductive surgery with or without HIPEC	Cisplatin
Cytoreductive Surgery (CRS) Plus Hyperthermic Intraperitoneal Chemotherapy (HIPEC) With Lobaplatin in Advanced and Recurrent Epithelial Ovarian Cancer	NCT03371693	Active, not recruiting	Ovarian Cancer, Epithelial Ovarian Cancer	112	HIPEC + CRS + CT, CRS + CT	Lobaplatin (HIPEC), Carboplatin, Paclitaxel, Gemcitabine, Liposomal Doxorubicin (HIPEC)
Secondary Debulking Surgery +/- Hyperthermic Intraperitoneal Chemotherapy in Stage III Ovarian Cancer	NCT00426257	Completed	Ovarian Cancer	242	Secondary debulking surgery, Secondary debulking surgery + HIPEC	Cisplatin (HIPEC), Carboplatin, Paclitaxel (IV)
Intraoperative Hyperthermic Intraperitoneal Chemotherapy With Ovarian Cancer	NCT01091636	Completed	Epithelial Ovarian Cancer	184	HIPEC	Cisplatin
Cytoreductive Surgery and HIPEC in First or Secondary Platinum-resistant Recurrent Ovarian Epithelial Cancer (HIPOVA-01)	NCT03220932	Not yet recruiting	Epithelial Ovarian Cancer	132	CRS + HIPEC, CT-BEV	Cisplatin, Bevacizumab
Efficacy of HIPEC as NACT and Postoperative Chemotherapy in the Treatment of Advanced-Stage Epithelial Ovarian Cancer	NCT03180177	Not yet recruiting	Epithelial Ovarian Cancer, Fallopian Tube Cancer, Primary Peritoneal Carcinoma	263	HIPEC, Interval debulking surgery, neoadjuvant chemotherapy, adjuvant chemotherapy	Paclitaxel, Cisplatin, Paclitaxel + Carboplatin (IV)
HIPEC for Platinum-Resistant Recurrent Ovarian Cancer (KOV-HIPEC-02)	NCT05316181	Recruiting	Epithelial Ovarian Cancer	140	HIPEC	Doxorubicin, Mitomycin
Hyperthermic Intraperitoneal Chemotherapy (HIPEC) in Ovarian Cancer (CHIPPI)	NCT03842982	Recruiting	Ovary Neoplasms, Ovarian Cancer, Ovarian Carcinoma	362	HIPEC	Cisplatin
Efficacy of HIPEC in the Treatment of Advanced-Stage Epithelial Ovarian Cancer After Cytoreductive Surgery (EHTASEOCCS)	NCT03373058	Recruiting	Epithelial Ovarian Cancer, Fallopian Tube Cancer, Primary Peritoneal Carcinoma	310	HIPEC, CRS, CT	Paclitaxel, Docetaxel, Paclitaxel + Carboplatin (IV)
A Randomized Prospective Trail of HIPEC in Recurrent Ovarian Cancer Patients With HRR Mutation	NCT04473339	Recruiting	Ovarian Cancer and Epithelial Ovarian Cancer with Homologous Recombination Repair (HRR) Gene Mutation	280	CRS, CRS + HIPEC	Lobaplatin
Hyperthermic Intraperitoneal Chemotherapy With Paclitaxel in Advanced Ovarian Cancer (hipecova)	NCT02681432	Unknown	Epithelial Ovarian Cancer	60	HIPEC, CRS only	Paclitaxel
Primary Cytoreductive Surgery With or Without Hyperthermic Intraperitoneal Chemotherapy (HIPEC) (OVHIPEC-2)	NCT03772028	Recruiting	Epithelial Ovarian Cancer	538	CRS + HIPEC	Cisplatin
Phase 3 Trial Evaluating Hyperthermic Intraperitoneal Chemotherapy in Upfront Treatment of Stage IIIC Epithelial Ovarian Cancer (CHORINE)	NCT01628380	Unknown	Ovarian Neoplasms	94	CRS, CRS + HIPEC	Cisplatin, Paclitaxel
Cytoreduction With or Without Intraoperative Intraperitoneal Hyperthermic Chemotherapy (HIPEC) in Patients With Peritoneal Carcinomatosis From Ovarian Cancer, Fallopian Tube or Primary Peritoneal Carcinoma (CARCINOHIPEC)	NCT02328716	Unknown	Peritoneal Carcinomatosis From Ovarian Cancer, Fallopian Tube Carcinoma, Primary Peritoneal Carcinoma	32	CRS, CRS + HIPEC	Cisplatin

Although drugs administered by HIPEC achieve better distribution, permanence, and penetration into the tumor tissue than systemic administration, this can only be performed once, immediately after CRS. Moreover, due to the physical properties of the liquids and the location of the inflow and outflow catheters, the exposure of the peritoneal surface to liquid drugs administered by HIPEC is incomplete (96). The use of an aerosol instead of a liquid drug solution could help overcome poor drug delivery. It has been widely shown that application of increased IP pressure increases drug uptake by tumor cells in both cases (97, 98) that in humans (99–103).

Based on these premises, a new IP delivery system called PIPAC (Pressurized IntraPeritoneal Aerosol Chemotherapy) was developed (104). PIPAC was first applied in humans in Germany in 2011 (105), and several European countries are now adopting it as a palliative therapy for patients with unresectable PC. PIPAC consists of drug nebulization into the peritoneum in the form of a polydisperse aerosol with an average droplet size of 25 µm at constant pressure and normotemperature. Unlike HIPEC, PIPAC is performed as a laparoscopic technique, is minimally invasive, and can be repeated several times after CRS. The aerosol nature of the drug solution used in PIPAC provides several advantages over other localized delivery techniques, such as more homogeneous tissue distribution of chemotherapeutics and higher drug concentration in the tumor microenvironment (104, 106). As mentioned earlier, the application of a constant pressure of 12 mmHg to the peritoneal cavity overcomes the pressure of the tumor interstitial fluid, resulting in higher local drug concentration and lower plasma levels of chemotherapeutics compared with IP or systemic catheter-based chemotherapy. The combination of pressure and aerosol also allows a more homogeneous distribution of droplets containing the active ingredient within the peritoneum, reaching exposed and even partially hidden surfaces, resulting in a prolonged antitumor effect with significant benefits on overall survival using a lower drug dosage (97, 98, 107).

Many parameters, such as aerosol droplet size, flow rate and solution viscosity, play a key role in the effectiveness of PIPAC, as they influence the physical and behavioral properties of the droplets. The optimal parameters required to achieve homogeneous drug distribution were studied by computational fluid dynamics modeling (108). The ideal droplet size was estimated to be between 1 and 5 µm, since gravitational forces had less impact on homogeneous drug distribution. However, commercial nebulizers are not able to reach those size, thus particles ranged between 30 and 50 µm are considered as a good compromise. Furthermore, higher flow velocity and low fluid viscosity are preferred because they are associated with both a reduction in particle diameter and an increase in spray cone angle, both of which promote homogeneous drug distribution (109). Several clinical trials have been performed since 2011 and more are ongoing. Phase I clinical feasibility studies of PIPAC found no signs of renal or hepatic toxicity, despite temporary impairment of portal and renal blood flows due to increased IP pressure. In addition, no signs of cumulative organ toxicity were found after repeated procedures of PIPAC (110). It has been generally observed that the dosage of doxorubicin, cisplatin and oxaliplatin administered via PIPAC is still far from the maximum tolerated dose (MTD) (107, 111–117), and in the case of oxaliplatin the dosage administered via PIPAC is approximately equal to 20 percent of the dose administered with HIPEC (107). The most recent ongoing study is still in phase of recruitment and aims to compare the efficacy of standard systemic treatments with IP aerosolization of cisplatin/doxorubicin combination (118). In this context, no systemic chemotherapy will be associated with the PIPAC procedure.

### 1.5 The emergence of ePIPAC

As described earlier, PIPAC is a viable alternative to conventional locoregional therapies, such as HIPEC and IPC, for patients with unresectable PC. Recent studies have shown that PIPAC can be improved by applying an electrostatic field during or after aerosolization of chemotherapeutic agents. Charged droplets precipitate electrostatically on tissues increasing cellular uptake of drugs (119). ePIPAC employs the same PIPAC equipment with the addition of an atraumatic stainless-steel brush electrode connected to a low-current generator. A weakly positively charged return electrode completes the system. Due to the collision of the emitted electrons with the aerosolized particles, the resulting negatively charged droplets are accelerated toward the peritoneum through the return electrode. The application of an electric field improves the spatial distribution of the droplets, increasing their ability to reach previously unreachable regions (119).

To date, there are only few studies in which the ePIPAC has been performed on patients. In the first human application of ePIPAC (120) only three patients with peritoneal metastases of hepatobiliary-pancreatic origin were enrolled, and although a positive response was observed, the obtained data were not sufficient to confirm the efficacy of the therapy. In 2019, ePIPAC was used in 48 patients (NCT03246321) with PM of different origin where it induced regression or pathology stabilization in about 50% of patients with no serious adverse effects (121). The safety and well tolerability of repeated ePIPAC procedures have been demonstrated in a retrospective cohort study published in 2021. The study included 69 patients treated with consecutive ePIPAC and oxaliplatin or cisplatin-doxorubicin combination in three centers from April 2019 to April 2020. About 76% of patients received concomitant treatment with systemic chemotherapy and in 38.5% and 53.8% of cases respectively, patients exhibited complete or greater histologic response (122). A new phase 1 research study (NCT05395910), initiated in October 2022 in Singapore and currently in the recruitment phase, aims to determine the safety profile and maximal tolerated dose of ePIPAC in combination with paclitaxel in pre-treated patients with PC. A summary of ongoing clinical trials involving PIPAC and ePIPAC for the treatment of ovarian cancer is summarized in **Table 2**.

**Table 2:** Clinical trials employing the use of PIPAC and/or ePIPAC for the treatment of disseminated peritoneal ovarian carcinoma.

Phase	Title	Identifier	Status	Conditions	Patients enrolled	Procedure	Drug
1/2	Pressurized Intraperitoneal Aerosol Chemotherapy (PIPAC) Applied to Platinum-Resistant Recurrence of Ovarian Tumor (PARROT)	NCT02735928	Unknown	Ovarian Epithelial Cancer Recurrent and Platinum-resistant	50	PIPAC	Cisplatin, Doxorubicin
1/2	Study of Efficacy and Safety of Laparoscopic Intra-abdominal Chemotherapy (PIPAC) Performed in Patients With Peritoneal Carcinomatosis From Colorectal, Ovarian, Gastric Cancer and Primary Peritoneal Tumors (PI-CaP)	NCT02604784	Completed	Peritoneal Carcinomatosis from Ovarian, Gastric and Colorectal origin	105	PIPAC	Cisplatin (15 – 30 - 50 - 67 - 88 - 93 - 100 mg/m^2^), Doxorubicin (3 - 6 - 10 - 13 - 18 - 23 - 30 mg/m^2^), Oxaliplatin (100 - 135 - 155 - 180 - 200 - 235 - 270 - 300 mg/m^2^)
1	PIPAC Nab-pac for Stomach, Pancreas, Breast and Ovarian Cancer (PIPAC nabpac)	NCT03304210	Completed	Peritoneal Carcinomatosis derived from Ovarian, Breast, Stomach and Pancreatic Cancer	20	PIPAC	Abraxane (nab-paclitaxel) (35 - 70 - 90 - 112.5 - 140 mg/m^2^)
1	A Study With Intraperitoneal Cisplatin and Doxorubicin in Recurrent Ovarian Cancer and Peritoneal Carcinomatosis (PIPAC-OV2)	NCT02475772	Completed	Ovarian Cancer	15	PIPAC	Cisplatin (7.5 - 11.25 - 15 mg/m^2^), Doxorubicin (1.5 - 2.25 - 3 mg/m^2^)
1	Pressurized Intraperitoneal Aerosol Chemotherapy (PIPAC) Associated With Systemic Chemotherapy in Women With Advanced Ovarian Cancer (PIPACOVA)	NCT04811703	Completed	Ovarian Cancer	15	PIPAC/IV chemotherapy	Cisplatin, Doxorubicin (PIPAC), Carboplatin, Paclitaxel (IV)
1	PIPAC for the Treatment of Peritoneal Carcinomatosis in Patients With Ovarian, Uterine, Appendiceal, Colorectal, or Gastric Cancer	NCT04329494	Recruiting	Ovarian, Uterine, Appendiceal, Colorectal, Gastric Cancer	49	PIPAC/IV chemotherapy	Cisplatin, Doxorubicin, Mitomycin, Oxaliplatin (PIPAC), Fluorouracil, Irinotecan, Leucovorin (IV)
1	International Registry of Patients Treated With Pressurized IntraPeritoneal Aerosol Chemotherapy (PIPAC) (PIPACRegis)	NCT03210298	Recruiting	Peritoneum, Pleural, Ovarian, Gastric, Appendix, Pseudomyxoma Peritonei, Colorectal, Pancreatic, Gallbladder Cancer	1000	PIPAC	n.d.
1	PIPAC With Nab-paclitaxel and Cisplatin in Peritoneal Carcinomatosis (Nab-PIPAC)	NCT04000906	Recruiting	Peritoneal Carcinomatosis	36	PIPAC	Nab-paclitaxel (7.5 - 15 - 25 - 37.5 - 52.5 - 70 mg/m^2^), Cisplatin
2	Intraperitoneal Aerosol High-pressure Chemotherapy for Women With Recurrent Ovarian Cancer (PIPAC-OV1)	NCT01809379	Completed	Recurrent Ovarian Cancer	69	PIPAC	Cisplatin, Doxorubicin
1	Pressurized Intraperitoneal Aerosol Chemotherapy (PIPAC) and Electrostatic PIPAC (ePIPAC) With Paclitaxel In Patients With Peritoneal Carcinomatosis	NCT05395910	Recruiting	Peritoneal Carcinomatosis	Estimated 36	ePIPAC	Paclitaxel

Both PIPAC and e-PIPAC may be useful in the treatment of peritoneal metastases. However, these techniques are still considered experimental treatments. Whereas PIPAC is typically used as a second-line treatment option for patients with recurrent peritoneal metastases after failure of previous systemic chemotherapy (123), e-PIPAC is still in its early stages and, despite promising results, has not yet been widely adopted in clinical practice. e-PIPAC is being studied for its safety and efficacy, and further research is needed before it can be suggested as a conventional treatment for peritoneal metastases (121).

The choice between PIPAC and e-PIPAC will likely depend on the clinical circumstances of the individual patient and the extent of peritoneal metastases. There are no absolute contraindications to either procedure, but patients with significant abdominal adhesions may not be suitable candidates for PIPAC or e-PIPAC. Adhesions, obliteration of the peritoneal space, organomegaly, bowel distension, or portal hypertension/cirrhosis have been found to affect the abdominal access procedure (124) and can generate difficulties of achieving even distribution of chemotherapy particles in the peritoneal cavity. In addition, patients with severe cardiovascular or pulmonary disease may not tolerate the procedure well because of the need for general anesthesia.

## 2 Exploring innovative drug delivery formulations as therapeutic approach for peritoneal carcinomatosis

Application of innovative drug delivery systems as micro and nanomedicines for the treatment of cancer has gained tremendous interest as they increase site specific drug delivery, attenuate drug toxicity, and protect drugs from rapid clearance (125). Since Doxil^®^, the first FDA-approved nanomedicine, more than 20 among lipid, polymer or inorganic nano- and micro- based drug delivery systems have become available in clinic for systemic administration in both therapeutic and imaging setting (126). Among them, 14 systems are currently employed in cancer treatment (127).

In the management of PC, the use of drug delivery systems can further ameliorate the efficacy of locoregional administration, since they can be designed to prolong the residence time in the peritoneal cavity and to target tumors, leading to a better toxicity/efficacy ratio (128). Despite an increasing number of preclinical and clinical studies are investigating the applicability of different delivery systems to the IP route (129, 130), this topic is young and, to date, there are still no clinically approved drug delivery systems for locoregional IP administration. However, different carriers are been tested, providing promising results. Polymeric and lipid nanocarriers with specific surface modifications have been conceived to improve tumor targeting, accumulation and residence time, whereas microparticles and hydrogel-based nanocomposites have been tuned to increase retention in the peritoneal cavity providing a controlled and sustained drug release.

**Figure 1:**
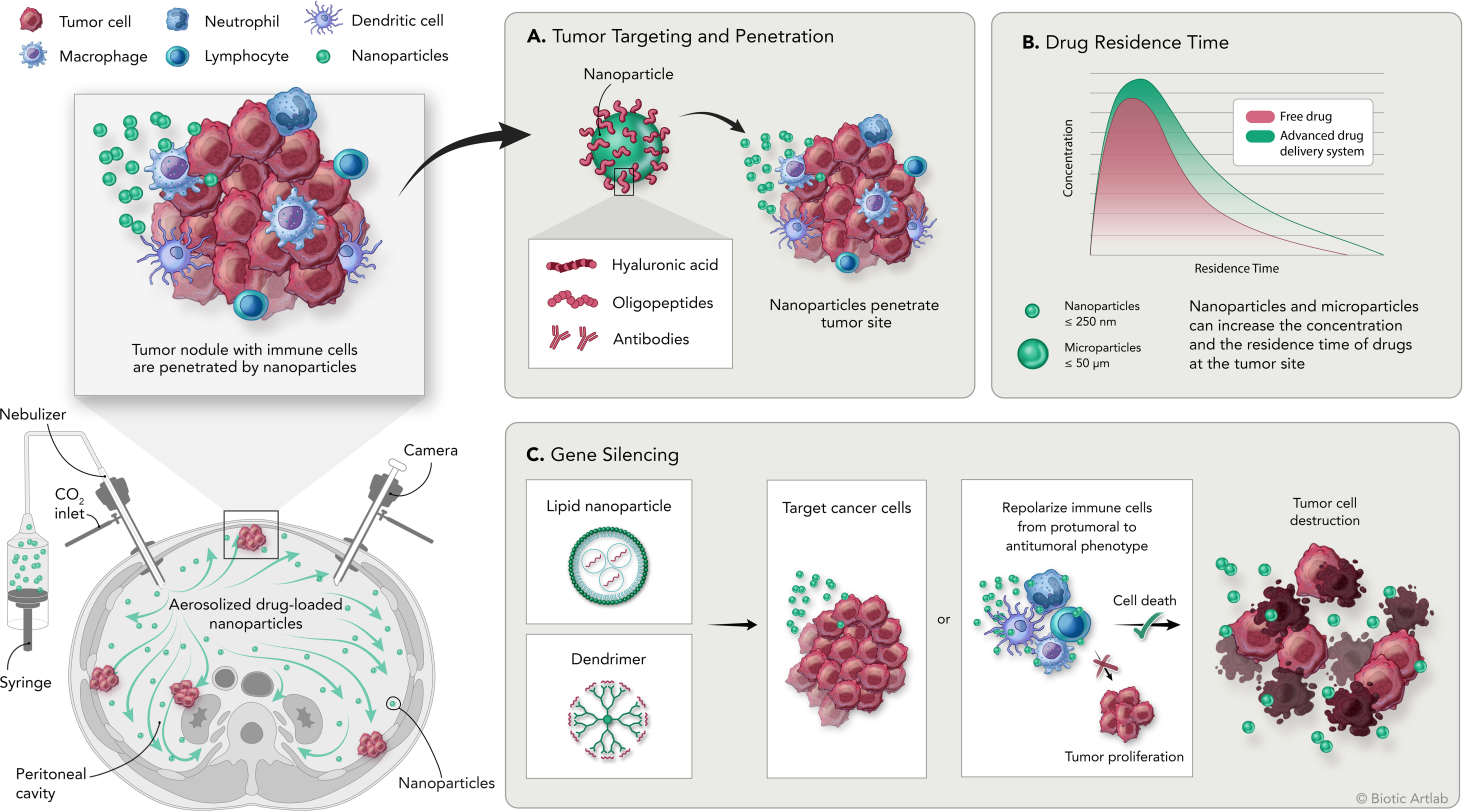
Overview of the PIPAC procedure and the application of nanomedicine for the treatment of peritoneal carcinomatosis. Bottom left: schematic representation of intraperitoneal aerosolization of nanoparticles by PIPAC; top left: schematic representation of tumor nodules with tumor infiltrating immune cell populations and delivered nanoparticles. **(A)** Nanoparticles can be modified by adding surface moieties of different origins to increase their targeting and penetration capabilities into the tumor. **(B)** Delivery systems are designed to increase the concentration and residence time of the drug at the tumor site compared with free drug. **(C)** Delivery systems designed to carry genetic material are used to induce silencing of specific genes on tumor or immune cells to promote direct or immune-mediated destruction of tumor cells.

Many of the features of these and others innovative drug delivery systems and their achievements are discussed in the following sections, summarized in **Table 3** and illustrated in **Figure 1**. Description of cell line characteristic cited in **Table 3** have been summarized in **Table 4**.

**Table 3:** Nanomedicines developed for intraperitoneal delivery.

Nanocarrier	Drug	Physicochemical characterization	Cell lines	Animal model	Studies outcome		Ref.
					*in vitro*	*in vivo*	
Ameliorating tumor targeting and penetration							
Hyaluronic acid-polyArginine nanoparticles (DACHPt-HA-pArg NPs)	DACHPt	Size: 249 nm Surface potential: -25 mV	SKOV3	Athymic nude female rats	(+) Better stability in ascitic fluids thanks to surface potential.	(+) When aerosolized (platinum dose 5 mg/kg), better tumor growth inhibition than free drug	(131)
Silica nanoparticles internalized into neural stem cells NSCs	Cisplatin	Size: 52 nm Surface potential: -17 mV	OVCAR8 and SKOV3	Female NOD-SCID mice	(+) Drug-loaded nanoparticles were toxic for NSCs only after 72 hours (IC_50 =_ 21.3 µM)	(+) Specific tumor targeting. (+) Better tumor penetration when associated to NSCs.	(132)
Polymeric expansile nanoparticles (eNPs)	Paclitaxel	Size: from 20 to 50 nm (at neutral pH) 250 nm (at acidic pH)	OVCAR3	Female nude mice	(+) Cytotoxicity not associated to eNPs alone (IC_50_ for paclitaxel loaded eNPs = 10 ng/mL).	(+) Selective localization in tumor areas. (+) Superior inhibition of tumor recurrence if compared with paclitaxel Cremophor EL^®^ formulations (paclitaxel dose 10 mg/kg).	(133)
RGD-decorated calcium phosphate nanoparticles	Doxorubicin	Size: 122.4 nm Surface potential: -2.3 mV	SKOV3 HK2	BALB/c-nu mice	(+) RGD peptide improves NPs internalization in SKOV3 cells (IC_50_ 11.13 mg/mL for RGD-decorated NPs vs IC_50_ 24.42 mg/mL for untargeted NPs). (+) Stronger tumor killing effect than on healthy cell line (HK2)	(+) Mice overall survival increased from 29 to 59 days (doxorubicin dose 10 mg/kg three times every five days). (+) No signs of drug-related toxicity.	(134)
iRGD-decorated polymersomes	Paclitaxel	Size: 233 nm Surface potential: -2.7 mV	PPC-1, M21, MKN-45P, CT26	Athymic nude mice, BALB/c mice	(+) Enhanced cytotoxicity for active-targeted polymersomes (paclitaxel concentration of treatment 100 nM). (+) Higher cellular uptake than not-targeted polymersomes	(+) Selective uptake in neuropilin-1 rich organs. (+) Reduced tumor burden (paclitaxel cumulative dose injected 7 mg/kg). (+) In CT26 model, reduction of ascites volume.	(135)
FRRG-doxorubicin nanoparticles (PNPs)	Doxorubicin (prodrug)	Size: 101 nm	H9C2, HDF, CDD-18Co, HeyA8, SKOV3, MC38, CT26, human ovarian tumor-bearing (POX) mice and human ovarian cancer patient derived xenograft (PDX) mice	BALB/c nu/nu and BALB/c mice	(+) Drug release specific to cancer cells. IC_50_ for SKOV3, HeyA8, MC38, CT26, H9C2, HDF and CCD-18Co cells were respectively 9.11µM, 5.06 µM, 8.98 µM, 5.2 µM, 111.36 µM, 111.72 µM and 135.8 µM.	POX model: (+) Lower PCI score than with saline or free drug (2.4 vs 13.8 and 6 respectively). (+) No associated systemic toxicity. PDX model: (+) tumor regression with homogenous drug tumor penetration and negligible organ toxicity. Both POX and PDX model used a doxorubicin dose corresponding to 5 mg/kg.	(136)
*Increase residence time*							
NanoOlaparib (lipid-based nanoparticles)	Olaparib	Size: 72 nm Surface potential: -30.5 mV	403 and 404 tumor line, (Brca2^-/-^, Tp53^-/-^, Pten^-/-^) 4306 and 4412 lines (K-ras^LSL-G12D/+^,Pten^-/-^)	Female NCr nude mice	(–) no IC_50_ difference from free drug (IC_50_ of NanoOlaparib for 4412, 404, 403 and 4306 cell lines were respectively 2.15 µM, 4.42 µM, 10.38 µM, 20.31 µM, while free olaparib IC_50_ were 2.49 µM, 3.43 µM, 10.94 µM, 19.57 and µM).	(–) Low retention time in the peritoneal cavity. (-) Daily IP administration not feasible due to systemic toxicity (NanoOlaparib injected dose 50 mg/kg).	(137)
NanoTalazoparib (lipid-based nanoparticles)	Talazoparib	Size: 71 nm Surface potential: +4 mV	mFT 3666, 3635,3665,3707 luc transfected cell lines ASC34, ASC54, ASC46 derived from ascitic fluid KURAMOCHI, OVSAHO	Female NCr nude mice	(+) IC_50_ values lower than IC_50_ of NanoOlaparib.	(+) Slow drug release. (+) 3/weekly administration sufficient to decrease tumor growth rate and ascitic fluid (NanoTalazoparib injected dose 0.33 mg/kg).	(138)
Bioadhesive polymeric nanoparticles BNPs (oxidized polylactic acid block–hyperbranched polyglycerol (PLA-HPG) copolymers)	Epothilone B	Size: 130 nm	Uterine serous carcinoma (USC)	Nude mice	(+) Lower IC_50_ than free drug after 72 hours of exposure.	(+) Bioadhesion with gradual drug release. (+) Two doses of Epothilone B were tested, 2.5 mg/kg and 0.5 mg/kg. Survival improvement (60% of treated mice alive at the end of the experiment).	(139)
Genipin-crosslinked gelatin microspheres (GP-MS)	Paclitaxel	Size: 50 µm	SKOV3 and OVCAR3	Female BALB/c Nu mice	(+) Drug-loaded-GP-MS were toxic for cells according to dose and exposure time. IC_50_ at 72 h and 168 h on SKOV3 and OVCAR3 increased from 8.6 and 9.5 nM to 4.9 and 7.1 nM respectively.	Two doses of paclitaxel tested: 7.5 mg/kg and 35 mg/kg. (+) Increase in median survival (from 33 days to 90 days). (+) Decrease in PCI score and ascitic fluid volume (comparison with ctrl and nab-paclitaxelVEG formulation)	(140)
Alginate-based cisplatin nanogel encapsulated in an *in situ* cross-linkable alginate-based hydrogel matrix	Cisplatin	Nanogel particles size: 10-30 nm	ID8-KRAS	C57BL/6 mice	(+) Lower cytotoxicity than free drug at 24 and 48 hours.	Cisplatin dose: 2 mg/kg and 10 mg/kg. (+) sustained drug release over a week. (+) increase in overall survival, reduction of VEGF expression and no observed adverse effects	(141)
Lipophilic nanocapsule loaded into PEG cross-linked hydrogel	Docetaxel	Nanocapsules size: from 174 to 250 nm Surface potential: -17 mV	–	Female BALB/c nude mice	(+) Hydrogel was stable upon dilution and ensure controlled nanocapsules release	(+) Nanocapsules incorporated in the PEG hydogel were retained in the IP cavity for 24 h after IP administration	(142)
Alendronate, calcium and cyclin-dependent kinase 7 inhibitor THZ1 self-assembled pH sensitive nanoparticles	Alendronate and THZ1	Size: 164 nm Surface potential: +12.4 mV	SKOV3, HK2, HMrSVS	BALB/c nude mice	(+) Intracellular uptake time-dependent. (+) Apoptosis induction through different mechanisms.	Administered dose: 10 mg/kg of nanoparticles. (+) Better % of apoptosis when THZ1 concentration increased. (+) Fluorescent NPs present in the tumor site 7 day after IP injection. (+) antitumor efficacy confirmed at 60 days after first treatment.	(143)
Tumor penetrating microparticles (TPM): Priming TPM (PLG 50:50 L:G) and Sustaining TPM (PLG 75:25 L:G)	Paclitaxel	Size: from 4 to 30 µm	SKOV3	Female athymic BALB/c Nu/Nu mice	–	Paclitaxel dose: 10 mg/kg. (+) Greater tumor targeting and therapeutic efficacy than paclitaxel-loaded cremophor-based formulations. (+) Better peritoneal cavity distribution for smaller particles.	(144)
*Gene silencing*							
Lipidoid siPARP1 nanoparticle	siPARP1	Size: 75 nm	*BRCA1* deficient ovarian cancer cell line	Nude mice	(+) Cells were efficiently transfected (65% of transfection after 24 h with 5 nM siRNA.	Total siRNA dose: 5 mg/kg. (+) PARP1 silencing confirmed by increased apoptosis, reduced tumor growth and increased mice overall survival.	(145)
HA-coated siPLK1 and sieIF3c loaded lipid-based nanoparticles	siPLK1 and sieIF3c	Size: 60 nm Surface potential: +5 mV	OVCAR8	Athymic nude female mice	(+) Synergistic antitumor efficacy of combined gene silencing. (+) Better internalization thanks to HA surface decoration.	Total siRNA dose: 1 mg/kg. (+) The combination of two siRNAs was more effective on mice overall survival (60% compared to 20 and 10% of single siRNA, PLK1 and eIF3c respectively).	(146)
Paclitaxel and siCD44 loaded polypropylenimine (PPI) dendrimer decorated with LHRH	siCD44 and Paclitaxel	Size: from 100 to 200 nm Surface potential: +1.10 mV	Human ovarian xenograft	Athymic nude mice	(+) Decoration with LHRH contributed to the antitumoral efficacy. (+) 10-fold decrease in cell viability compared to controls. 1.5-fold decrease in IC_50_ for formulate paclitaxel compared to unbound paclitaxel (from 55 µM to 34 µM).	Paclitaxel dose: 2.5 mg/kg. (+) Decrease in the invasiveness of malignant cells after CD44 suppression (+) Almost complete tumor eradication after 28 days.	(147)
Cisplatin and siDJ-1 PPI dendrimer decorated with LHRH peptide	siDJ-1 and cisplatin	Size: 145.2 nm Surface potential: +7.7 mV	ES-2 human ovarian clear cell carcinoma cells	Female athymic Nu/Nu mice	Preliminary *in vitro* studies were carried out to determine the siDJ-1 treating dose	siRNA dose: 50 µM in 0.5 mL volume. Cisplatin dose: 1.85 mg/kg. (+) After 35 weeks follow up, complete elimination of tumor mass without any recurrence.	(148)
siHuR-loaded fluorescent-labeled folic acid derivatized DNA dendrimers	siHuR	Size: 70 nm Surface potential: -28 mV	A2780, OVCAR5, OVCAR3, ID8-Fluc	C57BL/6 mice	(+) Tumor growth inhibition after HuR suppression.	siHuR dose: 3 µg/injection. (+) Tumor growth suppression and reduction in ascites formation. (+) Median life span increased from 29 to 43 days.	(149)
siTWIST-loaded hyaluronic acid conjugated mesoporous silica nanoparticles	siTWIST + cisplatin	Size: 120 nm Surface potential: +43.75 mV	F2, OVCAR8	Female NSG mice	(+) Cisplatin sensitization restored after TWIST suppression.	Nanoparticles dose: 2.5 mg/week. (+) 75% or 90% of tumor growth inhibition if compared with free drug or control group.	(150)
IRF5/IKKβ mRNA self-assembled to poly(β-amino ester) pre-functionalized with di-mannose poly glutamic acid	IRF5/IKKβ mRNA	Size: 100 nm Surface potential: +3.40 mV	ID8	Female albino B6 mice C57BL/6 mice for *ex vivo* studies.	(+) Reduction of the immune-suppressive macrophage population with increase in the M1-like macrophages fraction.	mRNA dose: 100 µg/mouse/week. (–) mRNA was also taken up by systemic circulation. (+) Tumor regression and immune activation with an increase in overall survival (142 days for treated mice vs 60 days for control groups)	(151)
Paclitaxel-pVSVMP loaded DPP nanoparticles	Paclitaxel and pVSVMP	Size: 197 nm Surface potential: +29 mV	SKOV3, A549, MDA-MB-231, MCF-7, CT26, B16	BALB/c nude mice	(+) Paclitaxel promoted transfection. (+) Reduced cell viability in presence of paclitaxel.	Paclitaxel, DPP and pVSVMP were administered at 1 µg/kg, 5 mg/kg and 0.2 mg/kg respectively. (+) Combination with paclitaxel enhanced gene transfection, while VSVMP antitumoral activity was confirmed.	(152)

**Table 4:** Description table of the cell lines cited in **Table 3**.

Cell line	Description
SKOV3	Human epithelial ovarian adenocarcinoma
OVCAR8	Human high grade serous ovarian carcinoma
OVCAR3	Human epithelial ovarian carcinoma isolated from malignant ascites
HK2	Healthy human tubular cell line from adult kidney
PPC-1	Human prostate carcinoma
M21	Human melanoma cell line
MKN-45P	Poorly differentiated human adenocarcinoma. Express wild-type p53; c-met oncogene amplification and E-cadherin promoter mutation.
CT26	Murine colorectal carcinoma cell from BALB/c mouse
H9C2	Embryonic rat cadiomyocytes
HDF	Human dermal fibroblast; skin cell line
CDD-18Co	Human fibroblast cell line isolated from normal colon tissue
HeyA8	Human epithelial low-grade serous ovarian cancer
MC38	Murine colorectal cancer
404	Murine tumor cell line with Brca2^-/-^, Tp53^-/-^, Pten^-/-^
403	
4306	Murine tumor cell line with K-ras^LSL-G12D/+^,Pten^-/-^
4412	
mFT 3666 luc	Murine fallopian tube cell lines developed from *Brca;Tp53;Pten* genetically engineered mouse model of high-grade serous ovarian cancer. They express *luciferase* gene for bioluminescent assays.
mFT 3635 luc	
mFT 3665 luc	
mFT 3707 luc	
ASC34	Murine tumor lines generated by culturing ascites collected from intraperitoneal murine tumor xenograft.
ASC54	
ASC46	
KURAMOCHI	Human high-grade serous ovarian cancer
OVSAHO	Human high-grade serous ovarian cancer
ID8	Murine surface epithelial ovarian cancer
ID8-KRAS	Murine surface epithelial ovarian cancer, oncogenic KRAS-transduced
HMrSVS	Healthy human peritoneal mesenchymal cells
ES-2	Human clear cell ovarian carcinoma
A2780	Human ovarian cancer cell line from an ovarian endometroid adenocarcinoma
OVCAR5	Human high grade serous ovarian cancer with possible gastrointestinal origins
ID8-Fluc	Murine epithelial ovarian cancer expressing *luciferase* gene for bioluminescent assays
F2	Human high grade serous ovarian cancer platinum resistant
A549	Human lung cancer
MDA-MB-231	Epithelial human breast cancer cell line
MCF-7	Human breast cancer cell line expressing estrogen, progesterone and glucorticoid receptors
B16	Murine melanoma

### 2.1 Drug delivery systems to improve tumor targeting and penetration

Passive accumulation via enhanced permeability and retention (EPR) effect or active targeting are the main drivers for delivery systems accumulation in solid tumors. The EPR effect has attracted great interest because of its success in preclinical animal models (153–155), but it has failed to demonstrate greater efficacy when studied in clinical setting (156, 157). Moreover, the advantage that the EPR effect offers in facilitating the accumulation of nanosystems in the tumor is relatively low and estimated at less than 2-fold compared with normal organs, and the resulting drug concentration is insufficient to treat most tumors (158). In the particular context of PC, tumor lesions differ in size and location and often have poor vascularization and perfusion, which prevent nanoparticles from take advantage of the EPR effect (159). IP delivery represents a more appropriate administration route because it exploits the irregularities and disorganization of mesothelial tissue caused by tumor cell infiltration, a mechanism that has been described as the main responsible of drug accumulation in tumor nodules following IP delivery (160, 161). However, a formulation developed for IV administration will not necessarily demonstrate better efficacy when administered via IP. An example is given by pegylated liposomal doxorubicin (PLD). The analysis of the pharmacokinetic curves of doxorubicin in patients receiving PLD via HIPEC following CRS, suggested a slow and variable absorption into the intraperitoneal tissues (40% of the administered drug was retained) with no advantages respect to the drug administered as such (162).

The addition of specific moieties to the surface of the nanoparticles facilitates their interaction via active targeting with specific molecules overexpressed at the tumor site. CD44 has already been extensively described as a suitable antigen for tumor targeting since it is overexpressed in a plethora of cancers as lung (163, 164), prostate (165), colon (166, 167), ovarian (168) and others (169). In several human ovarian cancer models, overexpression of CD44 is linked with cancer cells adhesion to peritoneal mesothelial cells (170). As the primary ligand of CD44, hyaluronic acid (HA) as an integral part of the nanocomposite structure has been shown to be effective in promoting preferential drug accumulation at the tumor site and enhancing cellular uptake (171, 172). The interaction between HA and CD44 expressed on SKOV3 cells mediates the internalization of polymeric nanoparticles generated by the electrostatic interaction between the positively charged amine groups of poly-arginine (pArg) and the carboxylic group of HA. Indeed, in the presence of free HA, internalization of the nanoparticles into tumor cells was significantly reduced and comparable to that obtained using a CD44-negative tumor (131, 173–175). Likewise, HA surface derivatization of a lipid-based nanoparticle containing a combination of two small interfering RNAs promoted their internalization in a OVCAR8 spheroid model, enhancing the effect of the therapy (146).

Active targeting is not limited to the use of small molecules as moieties for interaction with specific ligands/targets but can also employ larger molecules or even whole cells, as in the case of neural stem cells (NSCs). The tumor tropism of NSCs has been extensively studied, as has their ability to penetrate into hypoxic tumor (176–178). Nonporous, cisplatin-loaded silica nanoparticles were conjugated to the NSCs and optimized to avoid premature drug release that could have been toxic to the NSCs themselves. Comparison of IV and IP administration showed that active targeting was effective only after locoregional treatment. In addition, preliminary studies in OVCAR8 and SKOV3 tumor-bearing mice confirmed that NSC-internalizing particles administered IP had better tumor penetration ability than free drug or particles alone (132).

Tumor targeting can also be achieved through materials-based strategies, which take advantage of the intrinsic properties of the materials used rather than surface modifications.

The tumor microenvironment is often characterized by acidification due to glycolytic metabolism of tumor cells, hypoxia, and poor blood perfusion (179–182). These characteristics can be exploited by using polymers that react to the transition from the physiological to the lower tumor microenvironment pH by swelling and gradually releasing the encapsulated drug. An interesting example is represented by expansile nanoparticles (eNPs) characterized by a hydrophobic pH-cleavable protecting group which masks the hydrophilic linker (a triol group) and the polymerizing ending group (183). This cross-linked polymer is stable at physiological pH, while it starts to gradually hydrolyze from pH 6, thus releasing the encapsulated drug (183). In OVCAR3 tumor bearing mice undergoing debulking surgery to mimic clinical conditions, eNPs encapsulating paclitaxel (pax-eNPs) resulted more efficient compared to paclitaxel-Cremophor EL^®^ formulation in reducing tumor recurrence and biodistribution studies confirmed their specific tumor accumulation (133).

Active and material-based targeting strategies can also be integrated to develop formulations with multiple properties, as is the case with the combination of tumor homing peptides and pH-sensitive materials. Tumor homing peptides are oligopeptides up to 30 amino acids able to be specifically and efficiently internalized by tumor cells (184, 185). Some of the most widely used are the linear peptide RGD and its cyclic form iRGD. Both peptides consist of the amino acid sequence Arg-Gly-Asp that is known to recognize and bind αvβ3 integrins, which overexpression in tumors favors survival, proliferation and metastasis in cells of many different cancer models (186). In addition, the iRGD peptide also interacts with the neuropilin-1 receptor, increasing its permeability into the tumor tissue (187). RGD and iRGD have been conjugated to different types of drug delivery systems, such as polymeric nanoparticles (187), liposomes (184), dendrimers (188), hydrogels (189, 190), etc., showing promising results in the treatment of many types of cancer (191). These results have provided the rationale for implementing the use of these peptides in the treatment of PC by locoregional administration. In a study performed on SKOV-3 tumor bearing mice, RGD was conjugated to doxorubicin-loaded calcium phosphate (CaPO) nanoparticles, allowing the nanosystem to benefit from both RGD-induced active targeting and pH-dependent solubility of the CaPO scaffold. The resulting formulation presented a hydrodynamic size of 120 nm and a slightly negative surface charge, and was able to accumulate and release the drug into the tumor tissue. In addition, Ca^2+^ ions released from the particles accumulated in the cytoplasm of tumor cells causing mitochondrial dysfunction, increased cellular stress, and apoptosis. Once injected IP in SKOV3 bearing mice these particles induced a marked delay in tumor growth after two cycles of treatment increasing mice median overall survival from 29 to 59 days, without treatment-related toxicity (134).

The cyclic form of RGD, the iRGD peptide, has increased tumor penetrating abilities compared to RGD due to its ability to efficiently bind the transmembrane glycoprotein neuropilin 1 (NRP-1) in addition to α_v_β_3_ integrins. As well as α_v_β_3_, NRP-1 is often overexpressed in tumors, where it is implicated in multiple processes that promote tumor growth and invasiveness (192). Binding of iRGD with NRP-1 promotes its internalization, increasing the amount and rate of entry of the iRGD-bound nanosystem into cancer cells. Conjugation of iRGD peptide to a pH-sensitive polymersome made with POEGMA-PDPA and loaded with a fluorescent dye resulted in a compound (iRGD-PS-FAM) with a size of 233 nm and a slightly negative surface charge (-2.7 mV). Biodistribution studies showed that after IP administration on MKN-45P or CT26 tumor-bearing mice, iRGD-PS-FAM formulation was mainly detected in the tumor tissue. Furthermore, in the MKN-45P tumor model, colocalization of the formulation with blood vessels suggested that penetration of the compound into the tumors occurred from both the peritoneal cavity and systemic circulation (135). The same carrier loaded with paclitaxel showed better antitumor efficacy than Abraxane^®^, resulting in a significant reduction in the number of tumors in both MKN-45P and CT26 models (135).

Nanomedicines can also be designed to release the drug specifically at the tumor even without specific tumor cell binding. A short peptide substrate of cathepsin-B named FRRG (Phe-Arg-Arg-Gly), conjugated with doxorubicin and self-assembled in nanoparticles in presence of Pluronic^®^ F68 of has been used by Kim and colleagues to achieve tumor targeting via specific peptide cleavage and consequent disruption of the nanoparticles and release of doxorubicin. Cathepsin-B is a lysosomal protease constitutively expressed characterized by having either endopeptidase or exopeptidase functions at neutral or acidic pH respectively (193, 194), this enzyme is overexpressed by cancer cells and often associated with cancer progression (195). Conjugation between doxorubicin and FRRG gives rise to an amphiphilic molecule capable of self-assembly into a nanoparticle through π- π stacking and hydrophobic interactions; addition of Pluronic F68 improves *in vivo* stability, preventing immediate opsonization and particle elimination. Both IV and IP administration of the nanosystem showed good ability to accumulate in the tumor. Antitumor efficacy was confirmed in peritoneal human ovarian tumor xenograft (POX) and patient-derived xenograft (PDX) models, where treatment induced a two-fold reduction in PCI score and an increase in overall survival to more than 30 days compared with 19 days achieved with unformulated doxorubicin (136).

### 2.2 Drug delivery systems to increase residence time at the tumor site

Administration of chemotherapy via IP has been shown to increase drug concentration at the tumor site; however, its rapid elimination from the peritoneal cavity hinders therapeutic efficacy, which remains low. The use of nanomedicines designed specifically for IP administration is one strategy that can help overcome this problem. Still, the fate of nanoparticles after IP is as yet mostly unknown, and data on biodistribution are still limited.

To date, two main clearance mechanisms have been described for IP administration. Peritoneal absorption impacts molecules smaller than 20 kDa that, once diffused through capillaries, are drained into the portal vein, and eliminated. This size is typical of conventional chemotherapy treatments. Larger molecules and nanoparticles are drained through the lymphatic system: if the particles are larger than 500 nm, they are trapped in the lymph nodes, otherwise they can pass through the systemic circulation (196–198).

Nano-, micro- medicines and hydrogel-based nanocomposites, when properly designed, may improve the residence time of encapsulated drugs, and control their release over time. Several physicochemical features can contribute to increase residence time, as particle size, surface potential and the intrinsic properties of the material used (199).

Cationic liposomes and lipid-based nanoparticles, for example, have good peritoneal retention due to their interaction with the negatively charged peritoneal mesothelial cells, but there are also more prone to particle aggregation, which reduces lymphatic drainage (200).

A possible impact of surface charge on residence time has been found in the case of lipid-based nanoparticles loaded with olaparib or talazoparib. The two formulations, made with DPPC, cholesterol, DOTAP and DSPE-PEG_2000_, presented similar size around 70 nm and two different surface potentials, -30 mV for NanoOlaparib and + 4 mV for NanoTalazoparib. One hour after IP administration, majority of the olaparib was detected in the plasma, suggesting that the formulation was rapidly cleared from the IP cavity through systemic circulation. In contrast, 24 hours after the injection, 10% of NanoTalazoparib was still present in the IP cavity. The difference in clearance time is also associated with a different efficacy on tumor growth. In 404 tumor-bearing mice, NanoOlaparib treatment inhibited tumor growth only when administered daily, but caused serious side effects. However, increasing the dose and reducing the administration schedule resulted in loss of antitumor efficacy (137). IP administration of NanoTalazoparib over 3 times a week in mFT 3666 tumor-bearing mice resulted in a tumor volume reduction of more than 60%, compared to only 30% achieved by oral administration of the free drug (138).

Increased peritoneal residence time can also be achieved by using bioadhesive materials, that can help nanoparticles to interact with mesothelial cells and avoid fast lymphatic clearance (139, 201).

Polymeric nanoparticles made of polylactic acid block-hyperbranched polyglycerol (PLA-HPG) copolymers have been loaded with epothilone B, a potent microtubule-stabilizing agent targeting class III β-tubulin currently on phase II clinical trial for the treatment of ovarian cancer. Oxidation of vicinal diol groups on the surface of NPs induces their conversion to aldehyde groups that spontaneously react with amine residues of protein-rich surfaces including the peritoneal membrane and the tumor tissue (139). *In vivo* release studies performed on a xenograft model of uterine serous carcinoma, have confirmed that chemotherapy loaded on this bioadhesive formulation achieved higher drug concentration and longer peritoneal persistence leading to amelioration of mice overall survival and reduced drug-related toxicity (139).

While nanoparticles, because of their small size, need to firmly interact with the tumor microenvironment to increase their residence time in the peritoneal cavity, microparticles can simply take advantage of their size to be longer retained after IP administration (202–204). Specifically, when larger than 12 µm in size, particles can escape lymphatic duct drainage, thus avoiding being washed away and increasing its retention in the abdominal cavity (205). Also, because of their low surface area/volume ratio, the drug release of microparticles is slower than that of smaller particles, achieving better peritoneal distribution (205).

Microspheres cross-linked with genipin and loaded with paclitaxel were chosen for their biocompatibility (206). IP treatment of SKOV3-Luc-IP1 tumor-bearing mice showed an increase in median survival (from 33 days in the control group to 90 days in the treated mice), with a clear reduction in tumor burden, PCI score and ascitic fluid production (140).

Hydrogels, defined as three-dimensional, cross-linked networks of water-soluble polymers have been tested as IP administration for the treatment of PC, demonstrating antitumor efficacy (207–209). An *in situ* cross linkable hydrogel composed of alginate has been developed to effectively deliver cisplatin-loaded nanogel in disseminated PC of ovarian origin. This cisplatin-loaded nanogel was developed through a cross-linking reaction between chelating ligand and coordination metal and then loaded in the preparation of an alginate-based hydrogel. The size of the nanogel (10 to 30 nm) remained stable for 24 hours. *In vivo* antitumor efficacy was performed on ID8-KRAS tumor-bearing mice. Median overall survival increased by 10 days, with reduced VEGF expression and no signs of serious adverse effects (141). Another potential approach is represented by nanocapsule-loaded PEG cross-linked hydrogel. Nanocapsules were designed for hydrophobic drug loading, prepared using self-emulsification technique, and coated with HA through electrostatic deposition. The hydrogel matrix was based on poly-(ethylene glycol) thiol-maleimide cross-linking chemistry. Compared to thermosensitive hydrogels this preparation had better stability to dilution, often necessary in the case of preparation for peritoneal injections that require large volumes to be delivered. In addition, the IP administered hydrogel was retained in the peritoneum and able to release its load for up to one week (142).

In addition to the above-mentioned systems, the development of carrier-free nanodrugs has gained increased interest due to their easy manufacture and high drug load. Carrier-free nanodrugs can self-assemble via ionic contact, forming a polymer matrix with controlled-release features that favor high drug concentration at the target location and minimal systemic toxicity (210).

A novel pH-sensitive carrier-free nanomedicine, has been developed by combining the bisphosphonate medication alendronate, calcium ions, and THZ1. Alendronate is currently used in the treatment of osteoporosis (211), Paget’s disease of bone and bone metastases (212, 213). THZ1 is an inhibitor of cyclin-dependent kinase 7 (CDK7), an enzyme involved in the regulation of cell cycle progression and linked to increased transcription of oncogenes and increased proliferation rate of cancer cells (214). Alendronate and Ca^2+^ were assembled through coordination interactions, while self-assembly of THZ1 occurred through hydrophobic interactions. The presence of Ca^2+^ ions increased nanoparticles sensitivity to the acidic pH of the tumor microenvironment, favoring targeted drug release. In addition, as seen previously, their positive surface charge (+12.4 mV) facilitated interaction with mesothelial cells in the peritoneal cavity, increasing their retention and thus their residence time at the tumor site. Biodistribution studies performed on the SKOV3 tumor model showed that the nanoparticles were already homogeneously distributed in the peritoneal cavity one hour after injection and were still detectable one week later, with a preferential distribution in the tumor microenvironment. Efficacy studies confirmed the superior ability of the nanosystem compared to free alendronate or THZ1 alone in reducing both tumor growth and ascites volume, thus prolonging the median survival (143).

As mentioned earlier, the permeation of nanoparticles by EPR effect can be limited by inhomogeneous tissue permeability. This condition is strongly influenced by the high interstitial pressure that hinders the diffusion of the particles themselves. Chemotherapeutic agents such as paclitaxel or doxorubicin can be used to restore interstitial transport, as they stimulate apoptosis and amplify interstitial spaces, resulting in increased drug diffusion into tumor nodules. This priming mechanism can be incorporated into nanocarriers and combined with sustained drug delivery. This was achieved by combining poly-lactide-co-glycolide (PLG) copolymers with different rates of hydrolysis due to the 50:50 or 75:25 lactide:glycolide ratio. While PLG 50:50 hydrolyzes rapidly, PLG 75:25 degrades more slowly due to the lower number of glycolide monomers (215, 216). Both the formulations were loaded with paclitaxel thus allowing both rapid release of the drug resulting in a massive immediate action on the tumor and its priming, and a long-term release that sustains the chemotherapeutic action over time. Particle sizes in the µm range (4-30 µm) also helped reducing the clearance mechanism by further promoting peritoneal retention (217). Moreover, when compared with equivalent doses of active principle administered via cremophor-based preparation, these formulations demonstrated increased efficacy and lower general toxicity (144).

### 2.3 Delivery systems for the delivery of genetic material

Along with conventional therapeutic strategies, gene delivery technology has brought new, versatile and promising therapeutic approaches in biomedical research, especially with regard to cancer treatment. Pathological and dysfunctional states can be corrected by introducing into the cell with the necessary information to correct the expression of misleading proteins. This information is provided in the form of nucleic acid such as DNA, mRNA, siRNA, miRNA, and antisense oligonucleotide (218).

However, in most cases, genetic material cannot be directly injected into systemic circulation, as it would be easily degraded by enzymes (219) or recognized and eliminated by the immune system. Moreover, since genetic material exert its function inside the target cells, it need to safely cross numerous biological barriers, such as the endothelium and the extracellular and, in most cases, the nuclear membrane (220).To safely deliver genetic material to the cells both viral and non-viral vectors have been developed and non-viral nanoparticles, specifically lipid-based and polymer-based nanoparticles have emerged as a safer and more convenient delivery system compared to their viral counterpart (221–224).

In the case of PC in particular of ovarian origin, different pathways have been proposed as suitable target for gene silencing. For instance, siRNAs targeting the DNA repair machinery have been used to mimic the activity of PARP inhibitors and the administration of a lipidoid-siPARP1 nanoparticle in a *BRCA1-*deficient ovarian cancer mouse model successfully reduced tumor growth by causing the activation of apoptosis (145). Another lipid-based nanoparticle formulation was developed by Singh et al. to encapsulate a combination of two small interfering RNAs, eukaryotic translation-initiation factor 3c (eIF3c) and polo-like kinase-1 (PLK1), involved in the promotion of tumorigenesis and angiogenesis, and in the activation of early G2/M phase transition respectively. The strategy of simultaneously targeting two pathways has been chosen to improve efficacy of the treatment, whose effectiveness is often limited due to transitory effect of the silencing (225). Again, nanoparticles surface coating with HA moieties facilitated the internalization of the vector into the tumor cells. As expected, the combination of the two siRNAs has shown better therapeutic efficacy in increasing OVCAR8 bearing mice overall survival up to 60% compared to 20 and 10% of single siRNA, PLK1 and eIF3c respectively (146). CD44 has also been deeply investigated for its role in tumorigenesis and conferring resistance to treatments as indicated previously (226). Indeed, it has been shown that CD44 isoforms promote cancer cell survival and invasion by interacting with other molecules in the tumor microenvironment, such as fibronectin and hyaluronic acid, which promote cancer cell survival, adhesion, migration, and invasion. However, despite being a negative prognostic factor for ovarian cancer patients (227), CD44 widespread expression makes it a suitable target for nanoparticle-mediated therapy, as it can overcome drug resistance and improve drug delivery and accumulation in tumor tissue. At the same time, silencing its expression could bring significative advantages on tumor treatment. This hypothesis was tested in which a siRNA against CD44 was combined with paclitaxel and loaded in a dendrimer functionalized with the luteinizing hormone-releasing hormone (LHRH) peptide to confer the nanosystem targeting properties to cancer cells of gynecologic origin (228). *In vivo* studies on human ovarian xenografts confirmed that suppression of CD44 was responsible for increased tumor susceptibility to platinum-derived treatments leading to nearly complete tumor reduction (147). Few years later, the same research group has adopted a similar approach by silencing DJ-1 in the ES-2 metastatic human ovarian cancer IP injected in nude mice. DJ-1 is a protein expressed by more than 80% of human advanced ovarian carcinomas and linked to poor prognosis and chemotherapeutic resistance to platinum-based therapy in ovarian cancer (229). siDJ-1 was delivered using Poly(propylene imine) (PPI) generation 4 (G4) dendrimers coated with LHRH-modified PEG chains to confer targeting properties to the nanosized platform. Suppression of DJ-1 protein expression improved the antitumor efficacy of conventional therapeutic drugs, as this protein is involved in different pathways regulating oxidative stress as well as promoting survival, growth, and invasion of ovarian cancer cells (230–232). The combination of DJ-1 silencing, and cisplatin administration was sufficient to eradicate the tumor mass without any recurrence occurring in the following 35 weeks (148).

A different dendrimer-based nanosystem was used by Huang and colleagues to deliver siRNA for the silencing of human antigen R (HuR) protein to OVCAR5 human ovarian cancer injected in athymic mice. HuR is a human RNA-binding protein which main function is to stabilize mRNA to regulate gene expression (233) and that has been linked to bad prognosis in ovarian cancer patients. In this case, a novel developed double strand DNA-based dendrimer nanocarrier (3DNA, Genisphere^®^), functionalized with folic acid, was used to target tumor cells that highly expressed folate receptor α. HuR inhibition on ovarian ID8 tumor bearing mice resulted in decreased tumor growth and ascites formation, with consequent mice survival extension (149).

Another interest target for gene therapy is TWIST, a morphogenesis regulator gene implicated in the induction of epithelial-mesenchymal transition (EMT) in cancer cells. Acquisition of mesenchymal characteristics is a well-known mechanism associated to metastatic spreading and confers chemotherapeutic resistance to tumor cells (234). Silencing of TWIST mediated by siRNA loaded onto HA-conjugated mesoporous silica nanoparticles was effective in restoring mice cisplatin sensitivity in OVCAR8 model. Consequently, compared to control groups, ascites volume and tumor burden were significative reduced as well as number of metastases (150).

In addition to targeting cancer cells, gene delivery can be addressed to other components of the tumor microenvironment, such as immune cells, whose activity is often critical in determining tumor outcome. Elimination of immune suppressive cells as myeloid derived suppressor cells (MDSCs) or tumor associated macrophages (TAM) can lead to the restoration of T cells anti-tumor properties, and immune cell reprogramming or repolarization from a pro-tumor to anti-tumor status has been proposed as a tool to potentiate antitumor activity (235–237).

To achieve repolarization of TAM into macrophages with antitumor activity, Zhang at al. have exploited the function of IRF5 (interferon regulatory factor 5) that serves as a molecular switch controlling the pro- or anti-inflammatory polarization of macrophages and was chosen as target (238). They developed a nanosystem in which mRNAs encoding both IRF5 and its activating kinase IKKβ were self-assembled with a positive-charged poly(β-amino ester) (PbAE) polymer. The nanosystem was then pre-functionalized with di-mannose-poly glutamic acid (PGA) that is intended both to mask the residual positive charges, thereby stabilizing the nanocarrier, and to actively target CD206 TAM mannose receptor. *In vivo* studies conducted on ID8 ovarian cancer model confirmed tumor regression and activation of the immune response, while overall median survival passed from 60 days for control groups to 142 days for treated mice (151).

Another innovative way to avoid tumor progression is gene transfection with the vesicular stomatitis virus protein matrix plasmid (pVSVMP). In fact, the expression of vesicular stomatitis virus protein matrix leads to different mechanisms of destruction of the tumoral cell. Low dose paclitaxel was used in combination to improve gene transfection. The plasmid was loaded into a self-assembled cationic nanoparticle composed by paclitaxel, MPEG-PLA and DOTAP (P-DPP). A significative antitumoral efficacy on SKOV3 tumor model was confirmed, as well as the undeniable role of paclitaxel in enhancing the extent of growth inhibition (152).

### 2.4 Combination of nanomedicine and peritoneal aerosolization

The benefits that the application of nanomedicine can bring to the treatment of PC could be further enhanced by the combination with advanced peritoneal delivery techniques, such as PIPAC and ePIPAC. To evaluate the feasibility of the technique, Shariati and colleagues compared IV and IP injection of Lipofectamine™ MessengerMAX™ mRNA-containing lipoplexes with IP high-pressure nebulization (PIPAC). Biodistribution results confirmed a more homogeneous IP distribution of lipoplexes after PIPAC procedure. In addition, size, surface potential, mRNA complexation capacity as well as mRNA transfection efficacy of the commercial transfection tool were not affected by high-pressure nebulization (239).

However, nebulization processes may generate stress forces on the nanoparticles that could induce damage or deterioration of the delivery systems. Therefore, delivery systems must be appropriately designed to withstand the nebulization processes without being compromised and ensure effective and accurate delivery to the intended site. Homogeneous distribution and proper drug release depend greatly on the colloidal stability of the formulation, the maintenance of which after nebulization is closely related to its composition (240). Minnaert and coworkers compared the stability of two different aerosolized siRNA-encapsulating complexes, the lipid based Lipofectamine™ RNAiMax and a polycationic amphiphilic cyclodextrin, namely ADM70, on SKOV3. The nebulization process had a more important destabilizing effect on the ADM70 complex compared to RNAiMax, impairing its transfection efficiency. Moreover, the presence of ascitic fluid, typical of PC, dramatically decreased transfection efficiency of both systems but with a higher significance in the cyclodextrin-based complex, probably due to the formation of a protein corona around the nanosystem (241). Together with colloidal stability, size and surface charge of the nanoparticles have a massive impact on the residence properties of NPs in the peritoneal cavity, and both parameters need to remain stable during the nebulization process. In addition, the application of PIPAC rather than ePIPAC may require using different particles or their specific optimization. Positively charged curcumin loaded PLGA nanoparticles showed a better tissue penetration profile when associated with ePIPAC than a similar negatively charged PLGA formulation or PIPAC performed without an electrostatic field (242).

Viscosity is another property that can have a strong impact on nebulization results and must be considered especially when using hydrogels. Indeed, high viscosity can affect the angular cone of nebulization and thus the distribution of drugs in the peritoneum. By nebulizing five different concentrations of Pluronic F127 solution, ranging from 5 to 25% w/v, Braet and colleagues have proven how the increase of formulation viscosity was strongly associated to a dramatic decrease of the angle of aerosolization from 53.2° of the 5% w/v to 1° of the 25% w/v showing that further studies need to be done to optimize hydrogel-based nanomedicines for their application in PIPAC (243).

### 2.5 Clinical studies for the IP delivery of nanomedicine

To date only four clinical trials have been performed using nanoparticles for the delivery of drugs directly into the abdominal cavity via peritoneal infusion mediated by catheter (NCT00666991 and NCT00825201) or employing PIPAC (NCT03304210 and NTC05285358) (**Table 5**). NanoTax^®^ has been the pioneer compound used for IP administration with the double aim of offering a cremophor-free alternative to the IV administration of paclitaxel and increasing the reservoir of the drug in the peritoneal cavity. NanoTax^®^ is a nanoparticulate form of paclitaxel made by using supercritical carbon dioxide in combination with organic solvents in a process called supercritical fluid technology (244). This process results in naked, rod-shaped particles with narrow size distribution and mostly (≥95%) smaller than 1 μm (245). In 2008 the first multicenter open label dose-escalating phase I trial (NCT00666991) enrolled 21 patients to evaluate the toxicity and the pharmacokinetic profile of NanoTax^®^ by administrating a bolus injection through a previously implanted peritoneal catheter. Patients underwent six doses of NanoTax^®^, each one delayed of 28 days, ranging from a concentration of 50 to 275 mg/m^2^. The associated toxicity profile was comparable to the IV administration of paclitaxel with patients only experiencing low grade neutropenia, thrombocytopenia, or peripheral neuropathy, typical of paclitaxel IV treatment. Compared to IV administration, the concentration of drug measured in the peritoneal fluids was 450-2900 folds higher than plasma concentrations and remained elevated through the entire dose cycle due to extremely low peritoneal clearance, providing a marked benefit in tumor exposure intensity and duration of the treatment (246). A different approach was used in a second clinical trial started in 2009 (NCT00825201) where paclitaxel was administered IP encapsulated in a Cremophor-free formulation based on albumin nanoparticles (Abraxane^®^). Abraxane^®^ is currently approved by the FDA for IV administration for the treatment of breast, lung, and pancreatic cancer (247). Abraxane^®^, albumin-based nanocarrier (nab-paclitaxel) is an attractive system since, being physiologically present in human serum albumin can be safely considered nontoxic, non-immunogenic, biocompatible, and biodegradable (248). Due to its configuration, albumin can stably bind different drugs providing great advantages to their pharmacokinetic profile, moreover albumin mediates the drug uptake into the tumor cells by binding over-expressed receptors in tumor or endothelial cells (249). Additionally, techniques adopted for the formulation of albumin-based nanoparticles are highly reproducible and easily scalable, facilitating large scale manufacturing (248, 250). Abraxane^®^ was repeatedly administered via IPC to 27 patients affected by advanced peritoneal malignancies. When administered at maximum tolerated dose (MTD) of 140 mg/m^2^, drug plasma concentration was similar when compared to IV injection, however drug concentration in the peritoneal cavity was higher. These results were fundamental to set the basis for the study of Abraxane^®^ aerosolization in the peritoneal cavity. A multicenter dose-escalation phase I trial took place in 2017 (NCT03304210) to evaluate the safety of PIPAC-administered nab-paclitaxel in patients with unresectable malignancies and its results have been recently published (251). Five doses were evaluated (35-140 mg/m^2^), with a dose administration schedule of three times every four weeks, repeated for three cycles (130). Side effects were limited to the higher dosage with thrombopenia and neutropenia spontaneously recovering. Peripheral neuropathy with grade ≤2 was found only in patients with the highest dose. Results of this trial confirmed that PIPAC procedure is generally well tolerated in patients and showed that the combination of PIPAC and Abraxane^®^ has a favorable pharmacokinetics profile with an overall median survival of 10 months with 50% of patients surviving longer than 1 year. The latest clinical trial (NTC05285358) is currently ongoing on 12 patients to evaluate the safety of PIPAC nab-paclitaxel associated with systemic administration of gemcitabine and cisplatin.

**Table 5:** Clinical trials implementing the intraperitoneal delivery of nanomedicine for the treatment of peritoneal carcinomatosis.

Phase	Title	Identifier	Status	Conditions	Patients enrolled	Procedure	Drug
1	Pharmacokinetic, Safety and Efficacy Study of Nanoparticle Paclitaxel in Patients With Peritoneal Cancers	NCT00666991	Completed	Peritoneal Neoplasms	22	IP catheter	Nanoparticulate paclitaxel (NanoTax^®^) (50 - 82.5 - 125 - 175 - 225 - 275 mg/m^2^)
1	Intraperitoneal Paclitaxel Albumin-Stabilized Nanoparticle Formulation in Treating Patients With Advanced Cancer of the Peritoneal Cavity	NCT00825201	Completed	Ovarian Cancer, Peritoneal Cavity Cancer, Unspecified Adult Solid Tumor, Protocol Specific	27	IP catheter	Paclitaxel albumin-stabilized nanoparticle formulation (IP administration at day 1 - 8 - 15 for 28 days and then repeated)
1	PIPAC Nab-pac for Stomach, Pancreas, Breast and Ovarian Cancer	NCT03304210	Completed	Peritoneal Carcinomatosis, Ovarian Cancer Stage IIIB, Ovarian Cancer Stage IIIC, Ovarian Cancer Stage IV, Breast Cancer Stage IIIB, Breast Cancer Stage IIIc, Breast Cancer Stage IV, Stomach Cancer Stage III, Stomach Cancer Stage IV With Metastases, Pancreas Cancer Stage III, Pancreas Cancer Stage IV	20	PIPAC	Paclitaxel albumin-stabilized nanoparticle formulation Abraxane^®^ (35 - 70 - 90 - 112.5 - 140 mg/m^2^ every 4 week for 3 cycles)
1	Pressurized Intraperitoneal Aerosolized Nab-Paclitaxel in Combination With Gemcitabine and Cisplatin for the Treatment of Biliary Tract Cancer Patients With Peritoneal Metastases	NCT05285358	Recruiting	Distal Bile Duct Adenocarcinoma, Gallbladder Carcinoma, Intrahepatic Cholangiocarcinoma, Metastatic Malignant Neoplasm in the Peritoneum, Stage IV Distal Bile Duct Cancer AJCC v8, Stage IV Intrahepatic Bile Duct Cancer AJCC v8, Stage IV Intrahepatic Cholangiocarcinoma AJCC v8, Stage IVB Gallbladder Cancer AJCC v8	12	PIPAC	Gemcitabine + Cisplatin (IV on day 1, 3 and 5), nab-paclitaxel (PIPAC on day 3 of cycles 1, 3 and 5) repeated every 21 days up to 8 cycles

Although only few clinical trials have evaluated the nanoparticles IP administration feasibility, of which two (NCT03304210 and NCT05285358) employing PIPAC procedure, many interesting nanosystems cited in the previous paragraphs could be optimized for a future nebulization approach.

## 3 Conclusion

In summary, PIPAC and ePIPAC are gaining interest in the medical field as promising second-line therapeutic alternatives for patients with PC from EOC, while a plethora of novel drug delivery systems are being investigated to modify their pharmacokinetics and pharmacodynamics after administration.

Currently, the use of nanomedicine to improve tumor targeting and penetration is an active area of research and development, particularly for the localized treatment of PC. More research into the safety profile of nanosystems in comparison to current conventional treatment is required to validate their efficacy in treating cancer after IP or PIPAC. Biocompatibility, *in vivo* stability, drug loading efficiency in addition to targeting ability are the requirements that nanomedicines must meet to facilitate their translation from the bench to the bedside.

Despite the fact that many studies have been conducted using conceptually and technologically diverse nanoparticles, it is still difficult to predict which of them will be the most appropriate, safe, and effective in the treatment of this type of cancer because many of them are still in development and have only been tested in preclinical models. Furthermore, preclinical models of peritoneal carcinomatosis and PIPAC are still being developed and frequently lack complete and adequate characterization, such as from an immunological standpoint. A prerequisite that has yet to be fully addressed is the development of adequate models that depict the complexity of PC and enable the correct and repeated performance of the PIPAC method.

It should also be noted that clinical development will involve the transfer from small-scale to large-scale production, which may provide a significant challenge for some more complicated drug delivery systems. Liposomes and lipoplexes, as carriers of both chemotherapeutics and genetic material, are unquestionably a class of compounds that, because they are already in clinical usage, can enter the trial phase more quickly.

It is also important to remember that to date the current standard of care for the first-line treatment of ovarian cancer is based on the use of platinum derivatives alone, which are the most active chemotherapeutic drug class in this cancer. PIPAC’s research is currently focused on establishing a viable therapeutic line to address cases of recurrence that do not respond to conventional treatments. This includes not only recognizing situations in which this technique could benefit the patient, but also understanding the timing, dosages, and intervals of administration of PIPAC therapy. Currently, PIPAC is not considered a standard treatment option for ovarian cancer according to international guidelines. Therefore, it is premature to consider the use of PIPAC as first-line therapy for ovarian cancer, and no studies have been conducted in this area.

Then, innovation in PIPAC-associated nanosystems will be primarily related to the development of alternative therapies for refractory tumors. As a result, the development of nanomedicines capable of encapsulating drugs with therapeutic potential but difficult to administer in PIPAC due to chemical properties, such as hydrophobicity in the case of olaparib, or susceptibility to degradation as in the case of genetic material, may favor some nanosystems over others.

In conclusion, although this field is still young and much ground has yet to be covered there have been enormous breakthroughs and numerous novel ideas that have the potential to result in delivery methods able to improve the treatment of EOC derived PC.

## Author contributions

SB, SZ, and CG drafted the first version of the manuscipt. NB supervised the clinically relevant information. SZ, GL, and DK served as overall editors. All authors have contributed to the conception of the manuscript and revised it critically. All authors contributed to the article and approved the submitted version.

## Footnotes

1 World Health Organization. International Agency for Research on Cancer_The Global Cancer Observatory_Cancer Fact Sheets. 2018.

2 Cancer Research - Ovarian Cancer Survival.
